# HLA class I-restricted T cell epitopes isolated and identified from myeloid leukemia cells

**DOI:** 10.1038/s41598-019-50341-7

**Published:** 2019-10-01

**Authors:** Lydon Wainaina Nyambura, Alejandro Azorin Muñoz, Philipp le Coutre, Peter Walden

**Affiliations:** 1 Department of Dermatology, Venerology and Allergology, Clinical Research Group ‘Tumor Immunology’, Charité – Universitätsmedizin Berlin corporate member of Freie Universität Berlin, Humboldt-Universität zu Berlin, and Berlin Institute of Health, 10098 Berlin, Germany; 2 Medical Department, Division of Hematology and Oncology, Charité – Universitätsmedizin Berlin corporate member of Freie Universität Berlin, Humboldt-Universität zu Berlin, and Berlin Institute of Health, 10098 Berlin, Germany

**Keywords:** Immunosurveillance, MHC class I, Molecular medicine

## Abstract

Leukemia-associated antigens (LAAs) and HLA-I epitopes published previously have shown promise in inducing leukemia-specific T cell responses. However, the clinical responses are limited, and clinical effectiveness is yet to be achieved. Limitations, among others, being the LAAs themselves, the indirect approach to HLA-I epitope identification by reverse immunology, and the use of single or few LAAs and HLA-I epitopes, which limits the spectrum of inducible tumor-specific T cells. Use of a direct approach to identify naturally processed and presented HLA-I epitopes from LAAs, and higher numbers of antigens for T cell-mediated immunotherapy for leukemia may enhance clinical responses and broaden clinical effectiveness. In a prior study we used immunoaffinity purification of HLA-I peptide complexes from the differentiated myeloid tumor cell lines MUTZ3 and THP1 coupled to high-performance liquid chromatography tandem mass spectrometry (LC-MS/MS). From this we identified in the current study seven new HLA-I epitopes and the corresponding LAAs for myeloid leukemia. In comparison, the myeloid HLA-I epitopes reported here were generally stronger HLA-binders that induce stronger T cell responses than those previously published, and their source LAAs had higher immunogenicity, higher expression levels in myeloid tumors cells compared to normal hemopoietin and other major normal tissues, and more protein interaction partners, and they are targeted by CD8 T cells in CML patients. This study analyses and compares the LAAs and HLA-I epitopes based on various immunotherapeutic targets selection criteria, and highlights new targets for T cell-mediated immunotherapy for leukemia.

## Introduction

Cancer is the second leading cause of mortality worldwide and accounted for 8.8 million deaths in 2015^1^ with a projection of 13 million deaths in 2030. Leukemia, a group of hematological malignancies that encompasses acute myeloid leukemia (AML), chronic myeloid leukemia (CML), acute lymphoblastic leukemia (ALL), chronic lymphocytic leukemia (CLL) and other less common types, are among the 10 major cancers worldwide. They account for estimated 13.7 cases per 100,000 individuals per year and a five year survival rate of 61.4% (National Cancer Institute (NCI), Surveillance Epidemiology and End Results (SEER) 2008–2014; www.seer.cancer.gov).

Chemotherapy, radiotherapy, immunomodulatory drugs, proteasome inhibitors and hematopoietic stem cell transplantation (HSCT) have been used to treat hematological malignancies, though several of these are still incurable^2,3^. This is mostly due to the persistence of measurable minimal residual disease (MRD), which leads to high relapse rates^4,5^. So far, the only established immunotherapeutic approach is allogeneic stem cell transplantation, which has been shown to be essential in achieving long-term remissions^6–9^. However, it is associated with a high morbidity and mortality due to graft-versus-host activity of donor lymphocytes, and remains an option for only a fraction of patients^10–12^.

The alternative strategy to abolish MRD would be to use targeted T cell therapy, consisting of either adoptive transfer of T cells including use of chimeric antigen receptors (CARs) specific for leukemia-associated antigens (LAAs), or vaccination against LAAs^13,14^. Experimental leukemia immunotherapy using adoptive transfer of tumor antigen-specific T cells showed high efficiency but also severe toxicity in some patients due to previously unrecognized expression of target tumor antigen in vital organ^15^. On the other hand, a peptide based vaccine is an option and a number of T cell epitopes from LAAs have been identified based on gene expression profile analysis coupled to reverse immunology of computer-based T cell epitope prediction algorithms. Selected peptides based on this approach from Wilm’s tumor 1 (WT1)^7,14,16–22^, receptor for hyaluronan-mediated motility (RHAMM)^23,24^, telomerase reverse transcriptase (TERT)^25^, proteinase-3 (PRNT 3)^16^ and survivin^25^ alone or in combination, have already been tested in clinical trials. They have shown promising results in terms of induction of specific T cell responses as well as clinical responses in some patients^14,18,21,26–28^. However, broad clinical effectiveness is yet to be achieved. The limitations, among others, may be the choice of target LAAs, the indirect epitope identification, which is mostly based on overexpressed genes in leukemia patients compared to healthy individuals coupled to reverse immunology, and use of single or limited number of LAAs and epitopes, thereby limiting the spectrum of inducible tumor-specific T cell responses. The peptide identification criterion disregards the fact that mRNA expression reflects a distorted picture of the situation on the cell surface as detectable for T cells, and T cell epitopes can still be identified despite the absence of detectable respective mRNA^29^. This highlights the need for re-examination of LAAs and identification of new targets for T cell-mediated immunotherapy for leukemia like AML and CML.

In a prior study^30^, we used a direct method of target identification: immunoaffinity purification of human leukocyte antigen I (HLA-I) peptide complexes from the antigen-presenting immature and mature dendritic cells, and macrophages derived from the myeloid cell lines MUTZ3 and THP1 coupled to liquid chromatographic tandem mass spectrometry (LC-MS/MS). These cell lines were established originally from leukemia cells obtained from patients. In the current study we characterized HLA-I epitopes from potential LAAs (pLAAs), and compared the peptides and their source proteins with published HLA-I epitopes from established LAAs (eLAAs) based on experimental and predicted HLA-I binding affinities, ability to stimulate T cells, overall immunogenicity, gene expression profiles in leukemia and normal hematopoietic cells as well as major normal human tissues, and known protein interaction partners.

## Results

### Naturally presented HLA I epitopes from leukemia-associated antigens

**Figure 1:**
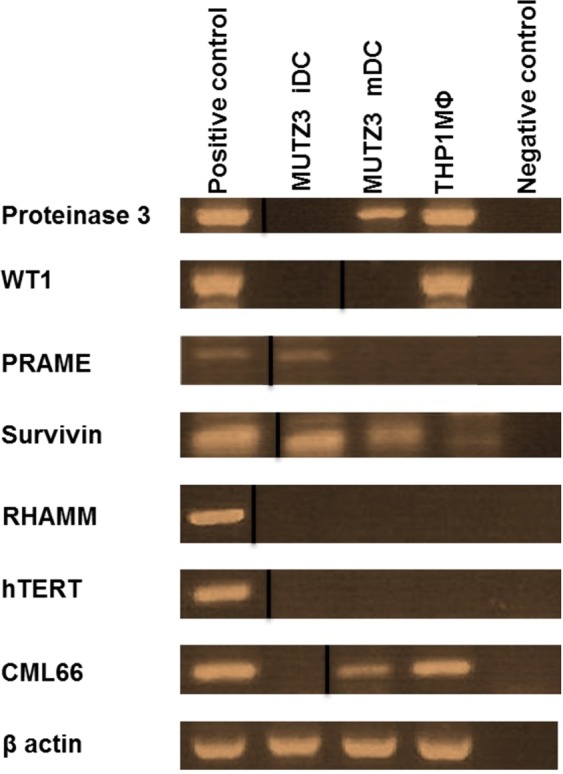
Expression of established leukemia-associated antigens (eLAAs) in MUTZ3 DCs and THP1MФ. RT-PCR showing expression of Proteinase 3, WT1, PRAME, Survivin, RHAMM, hTERT and CML66 in MUTZ3 DCs and THP1MФ. β actin was used as a control for the PCR reactions, a positive control (cDNA from cells positive for the respective antigen), and negative control (PCR minus cDNA) were included. All positive bands were of the predicted amplicon size. Proteinase 3 (363 bp), WT1 (380 bp), PRAME (154 bp), Survivin (129 bp), RHAMM (381 bp), hTERT (270 bp), CML66 (214 bp) and β actin (454 bp). The thick black lines on the gels delineate grouping of gels cropped from different parts of the same gel, or from different gels. Full length gels in Supplementary Fig. 1. Cropping: Proteinase, WT1 (Supplementary Fig. 1B), PRAME, Survivin (Supplementary Fig. 1C), RHAMM (Supplementary Fig. 1C,E), hTERT (Supplementary Fig. 1D,F), CML66 (Supplementary Fig. 1F) and β actin (Supplementary Fig. 1F). Expression of Proteinase and WT1 are also shown in Supplementary Fig. 1A, and CML-66 Supplementary Fig. 1D.

In our previous HLA peptidome analysis of the myeloid tumor cell lines MUTZ3 DCs and THP1MФ by LC-MS/MS^30^ the cells were lysed, MHC class I molecules isolated by affinity chromatography, and peptides extracted from the MHC molecules analyzed by LC-MS/MS. The sequences of a total of 975 HLA class I-bound peptides were identified from 852 source proteins^30^. From this data, HLA I epitopes from pLAA P141-MBOA7, P130-LARP1, P378-TRRAP, P207-PININ, P308-ROS1, P114-PSME3, P57-UHRF1 and P326-URP2 were identified (Table 1). These antigens but not the epitopes had been described previously for other malignancies and hematological indications^31–49^. In our HLA peptidome analysis, we had not identified any HLA I-bound epitopes from the eLAAs Proteinase 3, WT1, PRAME, Survivin, RHAMM, hTERT and CML66 that had previously been identified by reverse immunology of T cell epitope prediction algorithms (Table 2). To determine whether the lack of identification would be due to the lack of expression of the eLAAs in MUTZ3- and THP1- derived cells, RT-PCR was carried out using sequence-specific primers for the eLAA genes (Table 3). K562 and Molt4 cell lines were used as positive controls for RHAMM and hTERT respectively, and β actin as control for the PCR reaction. As shown in (Fig. 1 and Supplementary Fig. 1A–F), Survivin was expressed in MUTZ3 iDCs, MUTZ3 mDCs and THP1MФ, Proteinase 3 and CML66 in MUTZ3 mDC and THP1MФ, PRAME in MUTZ3 iDC and WT1 in THP1MФ. Expression of RHAMM and hTERT was not detected.

**Table 1:** HLA-A*02:01 epitopes derived from leukemia-associated antigens (LAAs) from MUTZ3 DCs and THP1MФ HLA-I peptidomes (potential leukemia-associated antigens (pLAA))^30^.

Protein	Peptide	Sequence	Peptide Source	Protein Role in Cancer (Reference)
MBOA7	P141-MBOA7	GLLPDVPSL	MUTZ3 iDC	^31– 33^
LARP1	P130-LARP1	ALPPVLTTV	MUTZ3 mDC	^34, 35^
TRRAP	P378-TRRAP	TLADLVHHV	MUTZ3 mDC	^36– 38^
PININ	P207-PININ	RLLEQKVEL	THP1MФ	^39– 41^
ROS1	P308-ROS1	HLVDEAHCLRL	THP1MФ	^42– 46^
PSME3	P114-PSME3	QLVDIIEKV	THP1MФ	^47^
URP2	P326-URP2	ALSNLEVKL	THP1MФ	^48, 49^
UHRF1	P57_UHRF1	TLFDYEVRL	THP1MФ	^71, 72^

*MBOA7*, Lysophospholipid acetyltransferase 7; *LARP1*, La-related protein 1; *TRRAP*. transformation/transcription domain-associated protein; *PININ*, 140 kDa nuclear and cell adhesion-related phosphoprotein; *ROS1*, proto-oncogene tyrosine-protein kinase ROS; *PSME3*, proteasome activator complex subunit 3; *URP2*, femitin family homolog 3; *UHRF1*, E3 ubiquitin-protein ligase UHRF.

**Table 2:** Clinically tested HLA-A*02:01 epitopes from established leukemia-associated antigens (eLAAs).

Protein	Peptide	Sequence	Immunogenicity and Clinical Relevance (Reference)
hTERT	P540-hTERT	ILAKFLHWL	^73, 74^
PRAME	P300-PRAME	ALYVDSLFFL	^75– 79^
WT1	P187-WT1	SLGEQQYSV	^80, 81^
RHAMM	P165-RHAMM	ILSLELMKL	^23, 24, 78, 82^
PRTN 3	P169-PRTN 3	VLQELNVTV	^14, 26^

*hTERT*, human telomerase reverse transcriptase; *PRAME*, melanoma antigen preferentially expressed in tumors; *WT1*, Wilm’s tumor protein; *RHAMM*, hyaluronan mediated motility receptor; *PRTN3*, Proteinase 3.

**Table 3:** RT-PCR primer sequences.

Gene	Primer Sequence (5′-3′)	Primer Binding	Melting Temp (°C)	Amplicon Size (BP)
PRTN 3	F: GCGGAGAACAAACTGAAC	333	65	363
	R: AGAAGTCAGGGAAAAGGC	696	65	
WT1	F: TAAAGGGAGTTGCTGCTG	307	65	380
	R: GGTGTCTTTTGAGCTGGT	687	65	
PRAME	F: TGAAAATGGTGCAGCTGG	679	65	154
	R: CGGGGAAATGTAGGAAGA	833	65	
Survivin	F: CCACCGCATCTCTACATT	47	65	129
	R: GAAGAAACACTGGGCCAA	176	65	
RHAMM	F: GCCTTAAGCAGTCTCTTG	808	65	381
	R: CTTTCAGCTTGTTCCTCC	1189	65	
hTERT	F: ATGAGTGTGTACGTCGTC	1644	65	270
	R: CGTAGTCCATGTTCACAATC	1914	66	
CML66	F: GTCAGTGCCACATTATGCTG	686	68	214
	R: CCTCCTTAGTACTGTCTTC	900	65	
ß-actin	F: AGGAGAAGCTGTGCTACGTC	722	59	454
	R: CTCGTCATACTCCTGCTTGC	1176	58

### Peptide binding by HLA-I

**Figure 2:**
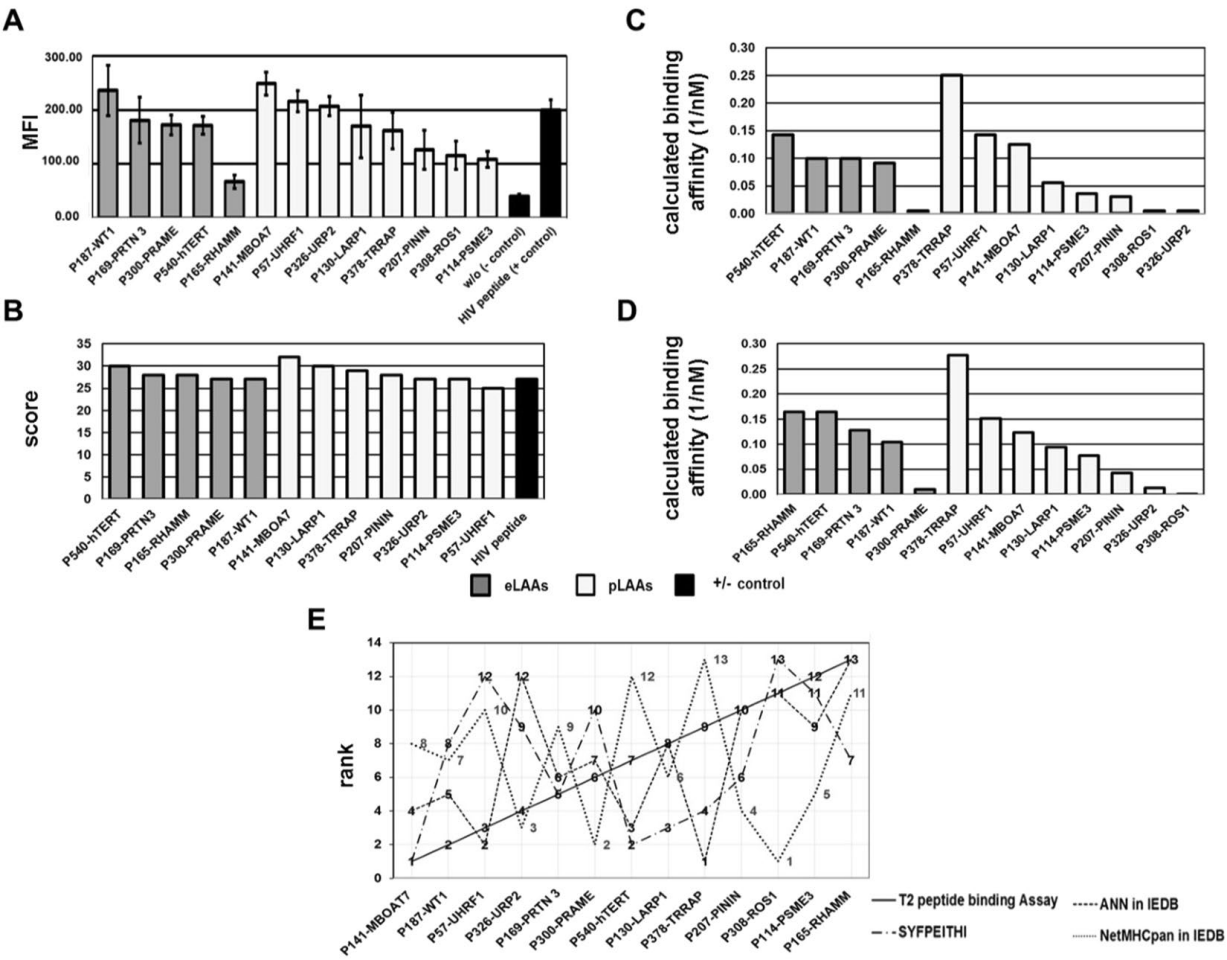
Peptide binding based on (**A**) experimental T2 cell line HLA A*02:01 stabilization assay (**B**) SYFPEITHI positional scoring matrix^61^ and (**C**,**D**) artificial neural networks-based affinity predictions in the immune epitope database IEDB^62,64^. Dark and light grey filling represent eLAAs and pLAAs, respectively. (**E**) Correlation of peptide ranking based on HLA-A*02:01 stabilization assay with the T2 cell line, SYFPEITHI scoring^61^ and artificial neural networks-based affinity predictions in the immune epitope database IEDB^62^.

Binding of HLA I-restricted eLAA epitopes previously identified from P300-PRAME, P540-hTERT, P165-RHAMM, P187-WT1 and P169-PRTN 3 by reverse immunology using T cell epitope prediction algorithms (Table 2), and of epitopes from P141-MBOA7, P130-LARP1, P378-TRRAP, P207-PININ, P308-ROS1, P114-PSME3, P57-UHRF1 and P326-URP2 identified by LC-MS/MS from the peptidomes of the myeloid cell lines MUTZ3 and THP1 HLA-I (Table 1) was determined with a HLA-A*02:01 positive TAP deficient T2 lymphoblastic cell line. The peptide binding evaluated by flow cytometry based on the mean fluorescence intensity (MFI) values from three independent experiments was in descending order P187-WT1 (236.16 ± 46.68) > P169-PRTN 3 (181.31 ± 42.47) > P300-PRAME (172.84 ± 18.59) > P540-hTERT (171.74 ± 16.67) > P165-RHAMM (67.40 ± 11.90) and P141-MBOA7 (249.31 ± 21.31) > P57-UHRF1 (215.82 ± 19.62) > P326-URP2 (207.33 ± 17.49) > P130-LARP1 (170.09 ± 57.83) > P378-TRRAP (161.77 ± 33.39) > P207-PININ (126.49 ± 35.89) > P308-ROS1 (116.25 ± 25.63) > P114-PSME3 (108.71 ± 14.64) for eLAAs and pLAAs HLA-I peptides, respectively. The MFI for without peptide (negative control) and P564-HIVPol (positive control) was 39.82 ± 4.16 and 199.91 ± 18.89, respectively. Except for P165-RHAMM, for all tested peptides the MFI values were twofold and above that of negative control (Fig. 2A).

Using the position-based amino acid scores in the epitope prediction algorithm SYFPEITHI the peptide ranking was P540-hTERT (30) > P169-PRTN 3, P165-RHAMM (28) > P300-PRAME (27) and P141-MBOA7 (32) > P130-LARP1 (30) > P378-TRRAP (29) > P207-PININ (28) > P326-URP2, P114-PSME3 (27) > P57-UHRF1 (25) for eLAAs and pLAAs HLA-I peptides, respectively (Fig. 2B). The score for P308-ROS1 was undetermined as no 11-mer prediction matrices for HLA-A*02:01 are defined with SYFPEITHI. The peptide binding affinities predicted by the ANN 4.0 algorithm in IEDB using a binding affinity threshold of IC_50_(500)nM was in the order P540-hTERT (0.14) > P187-WT1, P169-PRTN 3 (0.10) > P300-PRAME (0.09) > P165-RHAMM (0.01) for eLAAs HLA-I peptides and P378-TRRAP (0.25) > P57-UHRF1 > P141-MBOA7 (0.13) > P130-LARP1 (0.06) > P114-PSME3 (0.04) > P207-PININ (0.03) > P308-ROS1, P326-URP2, P165-RHAMM (0.01) for pLAAs HLA-I peptides (Fig. 2C). The peptide binding affinities predicted by the NetMHCpan 4.0 in IEDB using the same binding affinity threshold was similar to that of ANN 4.0 for pLAAs HLA-I peptides with profound differences in eLAAs HLA-I peptides prediction output (Fig. 2C,D). Binding affinity prediction for the P564-HIVpol peptide LLFGXPVYV was not possible by ANN 4.0 and NetMHCpan 4.0 in IEDB due to the undefined amino acid at position 5. Correlation of the peptide ranking was low among the four methods when the peptides were ordered from highest to lowest affinity binder in the T2 HLA I stabilization assay, despite similarities in the prediction output for pLAAs HLA-I peptides by ANN 4.0 and NetMHCpan 4.0 in IEDB (Fig. 2E).

### T cell responses to the established and peptidome epitopes

**Figure 3:**
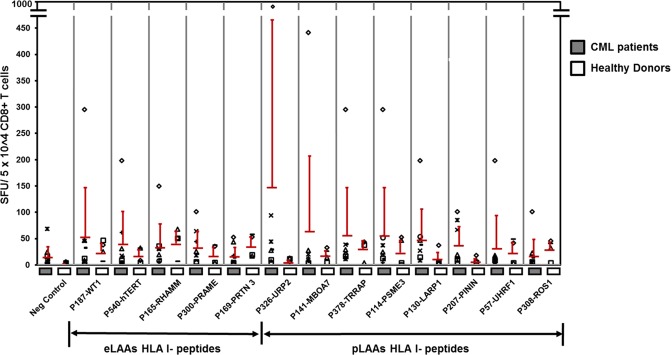
IFNγ ELISpot assay of PBMCs from 4 HLA-A*02:01 healthy donors (white bars) and 8 HLA-A*02:01 positive CML patients (dark grey bars). The cells were tested against the eLAA HLA-I peptides P540-hTERT, P300-PRAME, P187-WT1, P165-RHAMM and P169-PROTEINASE 3 previously identified by reverse immunology (Table 2) and the pLAA HLA-I peptides P207-PININ, P130-LARP1, P378-TRRAP, P308-ROS1, P114-PSME3, P326-URP2, P141-MBOA7, P57-UHRF1 identified from the MUTZ3 DCs and THP1MФ HLA-I peptidomes by LC-MS/MS (Table 1). PBMCs without peptides served as a negative control. Data represented as Spots Forming Units (SFU) per 5 × 10^4^ CD8+ T cells. Symbols represent individual data points.

To determine specific CD8+ T cell responses to eLAAs (Table 2) and pLAAs HLA-I peptides (Table 1), PBMCs from 4 HLA-A*02:01 healthy donors and 8 HLA-A*02:01 CML patients in chronic stage were stimulated for 7 days with the individual peptides with IL-2 addition at day 1 and 3, and analyzed for epitope-specific T cells by INFγ ELISpot assays as detailed in materials and methods. The frequencies of CD8+ T cells in the PBMC were determined by FACS. No major changes were seen in the frequencies in pre and post peptide-primed PBMCs (Supplementary Fig. 2). Based on Spots forming units (SFU) per 5 × 10^4^ CD8+ T cells the T cell responses from the 4 healthy donors in the ELISpot assay was below 41 ± 23 for both eLAAs and pLAAs HLA-I peptides (Fig. 3). For the 8 CML patients the order was P187-WT1 (54 ± 93) > P540-hTERT (39 ± 62) > P165-RHAMM (33 ± 44) > P300-PRAME (32 ± 31) > P169-PRTN 3 (15 ± 18) and P326-URP2 (147 ± 318) > P141-MBOA7 (63 ± 144) > P378-TRRAP (55 ± 91) > P114-PSME3 (47 ± 59) > P308-LARP1 (47 ± 59) > P207-PININ (36 ± 36) > P57-UHRF1 (30 ± 63), P130 > ROS1 (17 ± 32) for eLAAs and pLAAs HLA-I peptides, respectively (Fig. 3). P326-URP2 and P141-MBOA7 elicited the highest T cell responses and P169-PRTN 3 the lowest.

### Immunogenicity of the LAAs

**Figure 4:**
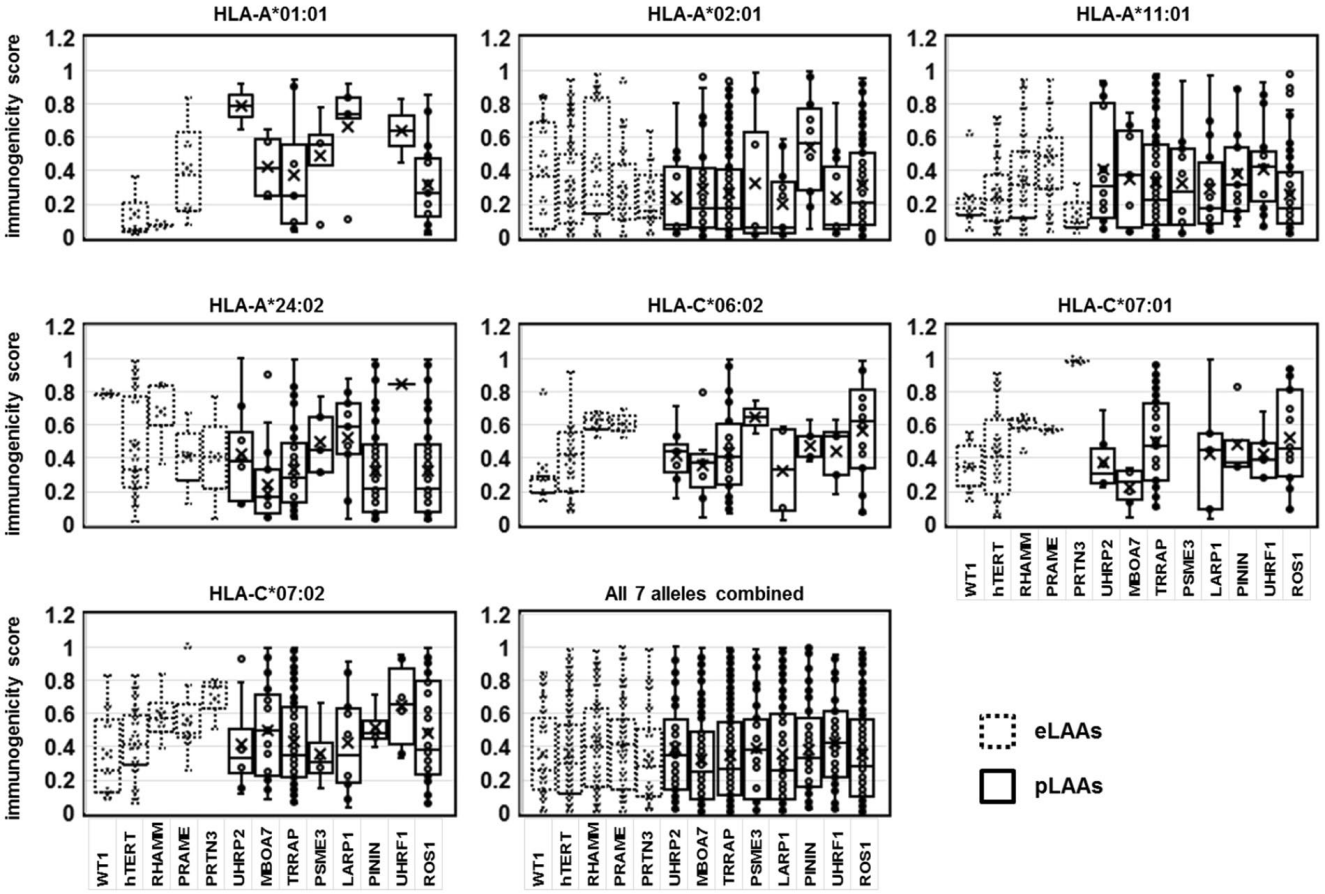
Immunogenicity of the LAAs for the most frequent HLA alleles. HLA-A*01:01, HLA-A*02:01, HLA-A*11:01, HLA-A*24:02, HLA-C*06:02, HLA-C*07:01 and HLA-C*07:02 that together represent more than 90% of the human population. The immunogenicity was determined with NetMHCpan 4.0 in IEDB^64^ using a binding affinity threshold of IC_50_ (500 nM). The immunogenicity scores are presented as 1/IC_50_ (500 nM) with values of 0 to 1 for low to high immunogenicity. Dotted and continuous line represent eLAAs and pLAAs, respectively.

The immunogenicity of eLAAs and pLAAs was assessed by reverse immunology epitope prediction applied for the most frequent HLA alleles HLA-A*01:01, HLA-A*02:01, HLA-A*11:01, HLA-A*24:02, HLA-C*04:01, HLA-C*06:02, HLA-C*07:01 and HLA-C*07:02 that together represent more than 90% of the human population. NetMHCpan 4.0 in the IEDB was used with a binding affinity threshold of IC_50_ (500 nM). The immunogenicity score is represented as 1/IC_50_(500 nM) and has a value of 0 to 1 for low to high immunogenicity. The immunogenicity of the LAAs varied depending the HLA allele (Fig. 4) and was generally highest for HLA-C*07:02 and lowest for HLA-A*02:01. The immunogenicity of both eLAAs and pLAAs for all the alleles was comparable, with median score of less than 0.4 for all LAAs.

### LAA gene expression in myeloid leukemia vs normal hematopoietic, and major normal tissues

**Figure 5:**
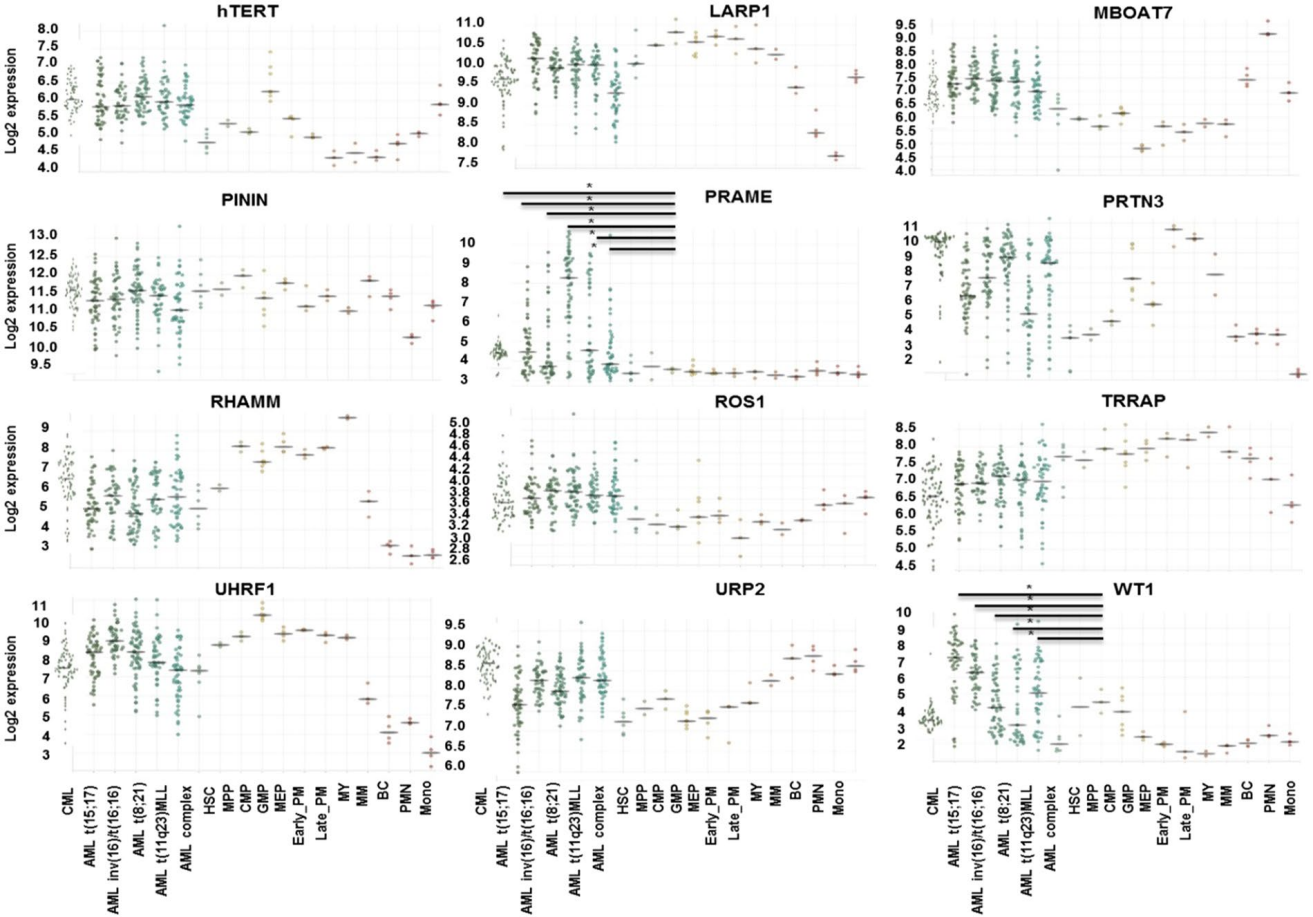
Gene expression profiles of the established leukemia-associated antigens (eLAAs) and potential leukemia-associated antigens (pLAAs) in CML, AML and normal human hematopoietic cells determined using Bloodspot^50^ and the datasets GSE13159 and GSE42519 for human CML and AML, and normal human hematopoietic cells, respectively.

**Figure 6:**
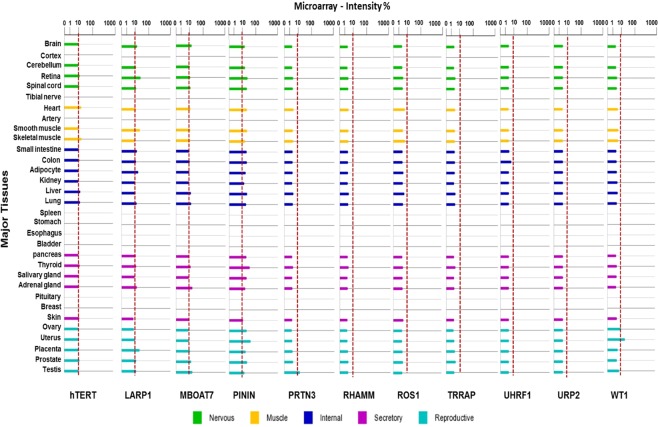
Gene expression profiles of the LAAs established leukemia-associated antigens (eLAAs) and potential leukemia-associated antigens (pLAAs) in normal human tissue analyzed and visualized using BioGPS (http://biogps.org/) and geneAtlas U133A gcrma datasets. The dotted line indicated low gene expression intensity cutoff of 10%.

The gene expression profiles of the LAAs (eLAAs and pLAAs) in CML, AML and normal human hematopoietic cells were compared using the Bloodspot database^50^ as detailed in material and methods. Only WT1 and PRAME expression was significantly higher in AML compared to normal hematopoietic cells *P < 0.05, and PRAME in CML (Fig. 5). The expression of ROS1 was elevated but not significant. The expression of the other LAAs in CML and AML cells was similar to those of normal human hematopoietic cells. The expression of the LAAs in CML and AML cells varied and, in decreasing order as per medium log2 expression values, was for CML in the order PRTN3 (10) > RHAMM (6.5) > hTERT (6) > PRAME (4.5) > ROS1 (3.6) > WT1 3.5 and PININ (11.5) > LARP1 (9.5) > URP2 (8.5) > UHRF1 (7.5) > MBOAT7 (7) > TRRAP (6.5) > ROS1 3.6 for eLAAs and pLAAs, respectively. For AML the order was PRTN3 (7.2) > TRRAP (7) > hTERT (6) > RHAMM (6) > WT1 (5) > PRAME (4.4) > ROS1 (3.8) and PININ (11) > LARP1 (10) > UHRF1 (9.2) > URP2 (8) > MBOAT7 (7.5) > TRRAP (7) > ROS1 (3.8) for eLAAs and pLAAs, respectively. The expression of the pLAAs LARP1, UHRF1, URP2 and MBOAT7 in AML was higher compared to all the eLAAs (Fig. 5). All the LAAs were expressed at low levels in all major normal human tissues based on a 10% gene expression intensity cutoff. Only hTERT, LARP1, PININ and WT1 were expressed beyond the 10% cutoff. Beyond this cutoff, hTERT was expressed in heart and skeletal muscle, LARP1 in brain, retina, smooth muscle, small intestine, adipocyte and placenta, PININ in all major tissues besides skin, and WT1 in uterus (Fig. 6).

### Protein interaction partners of the LAAs

**Figure 7:**
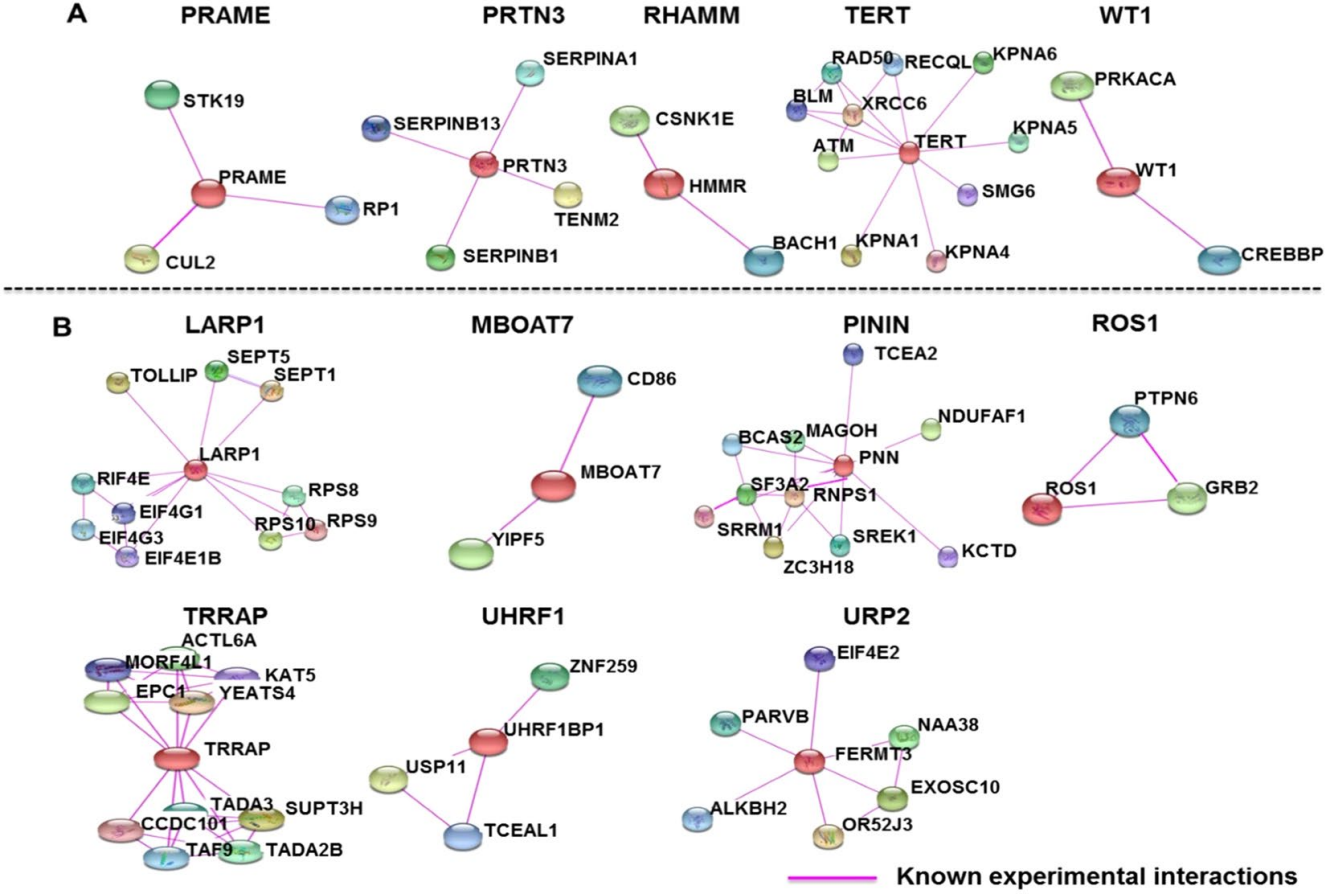
Known protein interaction partners of the LAAs established leukemia-associated antigens (eLAAs) and potential leukemia-associated antigens (pLAAs) determined using STRING version 11.0^66^ for *Homo sapiens* with a medium score of 0.400 and a cutoff of 10 interaction partners. (**A**) Protein interaction partners of the eLAAs. (**B**) Protein interaction partners of the pLAAs.

High numbers of protein interaction partners and the interaction of LAAs between and among them may indicate vital roles in leukemia. The known protein interaction partners of the LAAs (eLAAs and pLAAs) had been originally identified from experimental data obtained with a variety of biochemical, biophysical and genetic techniques. We applied STRING as detailed in materials and methods. WT1, RHAMM, ROS1 and MBOAT7 had only two known interaction partners, PRAME and UHRF1 3, PRTN3 4, URP2 6, and TERT, PININ, TRRAP and LARP1 10. (Fig. 7A,B). None of the LAAs were found to interact with each other, both at primary (directly via the first shell) (Fig. 7A,B) and at secondary level (indirectly via the second shell) (data not shown).

## Discussion

The eLAAs in Table 2 together with survivin and CML 66 have been described as ideal candidates for targeted immunotherapeutic strategy for leukemia especially AML as they are expressed in most leukemic blasts including leukemic stem cells, important for the leukemic phenotype, immunogenic and have shown clinical effective potential at peptide and protein level^51^. The identification of these eLAAs was based on the overexpression of their mRNAs in leukemia and the corresponding HLA-I peptides (Table 2) were identified by reverse immunology using T cell epitope prediction algorithms. In our previous analysis of HLA-I peptidomes of antigen presenting cell lines MUTZ3-derived immature and mature dendritic cells and THP1-derived macrophages by LC-MS/MS^30^ we didn’t identify any HLA-I peptides from these eLAAs. Despite the fact that the expression of the eLAAs, excluding RHAMM and hTERT, were detectable in MUTZ3 DCs and/or THP1MФ. This tallies with previous studies that have shown that mRNA gene expression does no translate directly into HLA epitope presentation, and reflects a distorted picture of the situation on the cell surface as detectable for T cells^29^. In fact, HLA-I peptides have even been identified without detectable mRNA expression of their source proteins^29^.

The eLAAs and HLA-I epitopes have shown promising results in terms of induction of specific T cell responses, however, with limited clinical responses^14,18,21,26–28^. The restrictions may be the choice of LAAs mostly based on mRNA gene expression profiles, the indirect HLA-I epitope identification criteria, and the use of single or limited number of LAAs and HLA-I epitopes, which limits the spectrum of inducible tumor-specific T cell responses. The use of a direct approach to identify HLA-I epitopes from pLAAs and higher number of LAAs and HLA-I epitopes for targeted immunotherapy for leukemia could enhance clinical effectiveness.

In a prior study, we used immunoaffinity purification of HLA-1 of the antigen presenting lines MUTZ3-derived immature and mature dendritic cells and THP1-derived macrophages together with LC-MS/MS of the peptides extracted from the HLA-I^30^. In the current study, we identified HLA I-presented epitopes from these HLA I peptidomes of antigens that had been described for other malignancies and hematological indications^31–49^. We analyzed and compared the LAAs and HLA-I peptides in Table 2 (epitopes from eLAAs) with those in Table 1 (epitopes from pLAA) based on their experimental and predicted HLA-binding affinities, immunogenicity, expression of their source proteins in leukemic cells vs normal human hematopoietic cells and normal major human tissues, and their protein interaction partners. All these analyses and comparisons are important to assess the suitability of LAAs and HLA-I epitopes as immunotherapeutic targets in leukemia, which should contain epitopes with high affinity for HLA, be highly immunogenic for induction of tumor-specific CD8 T cells, and be highly interconnected with essential pathways so that they cannot be down-regulated without damage to vital processes.

Though all HLA-I peptides had high HLA-binding affinities based on the T2 cell HLA-A*02:01 stabilization assay, peptides P141-MBOAT7, P378-TRRAP and P57-URP2 from the pLAAs (Table 1) had higher binding affinities than P300-PRAME, P540-hTERT, P165-RHAMM and P169-PRTN 3 from the eLAAs (Table 2). By SYFPEITHI epitope prediction, all the peptides had high score between 25 and 30. Using ANNs in IEDB, P378-TRRAP, P57-UHFR1 and P141-MBOAT7 from pLAAs ranked higher than the P540-hTERT from eLAAs (Fig. 2). There was no correlation in the ranking of the 12 peptides with respect to their HLA binding among the four methods, T2 HLA stabilization assay, SYFPEITHI, and NetMHCpan 4.0 and ANN 4.0 in IEDB (Fig. 2). The lack of correlation may have different reasons. Of the four methods, only the HLA stabilization assay is an experimental assay that, although not measuring affinity directly, yields values strongly correlated with binding affinity. The prediction algorithms are based on different types of data and have different designs. SYFPEITHI as a position-specific scoring matrix basically computes likelihood of binding of a peptide to a given HLA based on the statistics of the amino acids at the different sequence positions, which ignores the sequence contexts. The ANNs, as other machine learning approaches, can in principle capture information on the sequence contexts but are based on training data sets for binders and non-binders. The positive training sets usually differ between the different ANNs and reflect the composition and histories of the data: training data with exclusively experimental peptidome sequences may result in different predictions than training data with a high proportion of predicted epitopes. Negative training data are usually difficult to come by and often binders for a different HLA allele are used. Moreover, ANNs may work with different data formats such as peptide sequences or binding kinetics. Notwithstanding the lack of strict correlation, P141-MBOAT7 and P378-TRRAP was highest ranked among the four methods and P165-RHAMM and ROS1 were lowest.

In ELISpot assays for the frequencies of LAA epitope-specific T cells, the responses were higher in CML patients compared to the healthy controls, dominated by HLA-I peptides from the pLAAs, and P187-WT1 and hTERT from eLAAs (Fig. 3). Even though there was no correlation between the peptide binding affinities and T cell responses, maybe due to limitation of peptide binding affinity prediction approaches mentioned, in tendency HLA-I peptides from pLAAs were stronger binders with higher T cell responses than those from eLAAs. Since *in vitro* studies often do not translate into clinical effectiveness^52,53^, a comparison of the HLA-I peptides from eLAAs and pLAAs in a clinical study would be vital.

The sources of LAAs are equally important as the HLA-I peptides. In this regards, the predicted overall immunogenicity of the LAAs for the seven most frequent alleles combined was high and comparable between the eLAAs and pLAAs (Fig. 4). All the LAAs appear suitable as therapeutic targets in leukemia patients from the general human population.

mRNAs of all the LAAs were expressed in CML and AML cells, but the pLAAs at higher levels (Fig. 5). Comparing the gene expression profiles between leukemia cells and normal human hematopoietic cells, only WT1 and PRAME expression was significantly higher in leukemia cells compared to normal human hematopoietic cells, in CML only PRAME (Fig. 5). In addition, TERT, WT1, LARP1 and PININ and were found to be highly expressed in some normal human tissues (Fig. 6). Similar observations were reported for previous studies for TERT and WT1^54–56^. For leukemia-targeted immunotherapy, especially peptide-based vaccines and TCR-based CARs, differential gene expression may only play a secondary role in target selection. Gene expression levels of the antigens do not correlate with the presentation of the epitopes from these antigens on the cell surface as detectable for T cells^29^. For instance, though WT1 is expressed in normal tissues such as gonads, kidney and normal hematopoietic cells^54,55^, it has been used in clinical trials for targeted immunotherapy especially peptide-based vaccine for leukemia without any serious immunologic adverse events being observed^14,21^.

Cancers can switch off genes/lose antigens as an immune escape mechanism^57–59^. As predicted from protein network studies, they are more likely to switch off antigens with low number of protein interaction partners. The higher the number of protein interaction partners a LAA has, the more vital the LAA may be for cancer cell survival. Such LAA could be a more suitable immunotherapeutic target for leukemia. In this regard, PININ, TERT, TRRAP and LARP1 had the highest number of interaction partners, PRTN3 and URP2 intermediate numbers and WT1, RHAMM, ROS1 and MBOAT7, PRAME and UHRF1 the least (Fig. 7). Furthermore, no LAAs were found to interact with each other, both at primary level (directly) and at secondary level (indirectly).

In conclusion, the results of this study highlight new potential targets for T cell-mediated immunotherapy (therapeutic vaccine and TCR-based CARs) for leukemia, and the analysis and comparative data may allow for more informed selection of LAAs and HLA-I epitopes. However, comparative studies in a clinical setup are necessary. Furthermore, though mass spectrometry (MS)-based immunopeptidomics approaches yield a large and relatively unbiased portrait of the population of processed and presented peptides, technological limitations such as low sensitivity may need to be addressed to allow for identification of more new potential targets for T cell-mediated immunotherapy.

## Materials and Methods

### Cell lines

The acute myeloid leukemia cell line MUTZ3 (DMSZ GmbH, Braunschweig, Germany) and acute monocytic cell line THP1 (ATCC TIB-202) were cultured and differentiated to MUTZ3-derived immature and mature dendritic cells (MUTZ3 iDC and MUTZ3 mDC), and THP1 macrophage (THP1MФ) respectively, as detailed in supplementary methods. The T cell leukemia cell line Molt-4 (DSMZ, Braunschweig, Germany) and the TAP-deficient lymphoblastic cell line T2 (from Peter Cresswell, Yale, New Haven, CT, USA) used in the HLA-A*02:01 peptide binding affinity assays were cultured in DMEM or RPMI 1640 medium (Invitrogen, Karlsruhe, Germany) supplemented with 10% heat-inactivated fetal calf serum and 1% penicillin/streptomycin at 37 °C under 8% CO_2_. MUTZ3 iDC, MUTZ3 mDC, THP1MФ, K562 and Molt-4 cells were harvested by 10 min centrifugation at 800 × g, shock-frozen with liquid nitrogen and stored at −140 °C. K562 and Molt-4 were used for total RNA isolation and MUTZ3 iDC, MUTZ3 mDC and THP1MФ for pHLA extraction or total RNA isolation.

### PBMCs from healthy volunteers and CML patients

The clinical material was used with approval by Charité ethics committee (Approval No. EA1/222/14 and EA1/026/14) and written informed consent by the patients and volunteer donors. All methods and procedures were performed in accordance with the relevant guidelines and regulations including the handling of primary clinical materials, and anonymization and processing of patient-related information and data. HLA typing was carried out by the HLA typing laboratory, Charité - Universitätsmedizin Berlin. Peripheral blood of 4 HLA-A*02:01-positive healthy volunteers and 8 HLA-A*02:01-positive chronic myeloid leukemia (CML) patients was used to isolate PBMCs by density centrifugation using Ficoll Paque (Biochrom, Berlin, Germany) and cryopreserved in FCS (Biochrom, Berlin, Germany) with 10% DMSO (Pierce, Illinois, USA) at −140 °C. For *in vitro* priming, PBMCs were pulsed with 10 µg/ml of each peptide in ExVivo 15 serum-free medium (Biowhitaker, Belgium) at 37 °C and 8% CO_2_. 50 U/ml of recombinant human IL-2 (Chiron, Munich, Germany) was added to the cultures on day 1 and day 3. On day 8 the primed PBMCs were harvested, washed with PBS (Gibco, Grand Island, NY, USA) and counted before analysis in INFγ ELISpot assays detailed in supplementary methods and by flow cytometry.

### Gene expression in THP1MФ and MUTZ3 DCs using RT-PCR

Total RNA was extracted from THP1MФ, MUTZ3 DCs, K562 and Molt-4 using Nucleospin RNA II purification kit (Macherey-Nagel, Duren, Germany) as per manufacturer’s instructions. 500 ng of DNase-treated RNA was used to prepare cDNAs using superscript III Reverse Transcriptase (Invitrogen, CA, USA). RT-PCR was carried out with 1 µl of cDNA with PRTN3-, WT1-, PRAME-, Survivin-, RHAMM-, hTERT-, CML66-, β actin-sequence specific forward and reverse primers (Table 3). PCR conditions were initial denaturation at 96 °C for 4 min, followed by 34 cycles of 40 sec at 95 °C, 1 min at 55 °C to 68 °C, 40 sec at 72 °C, and a final extension step of 10 min at 72 °C. PCR products were analyzed by 1% agarose gel electrophoresis. Positive controls for RHAMM and hTERT were K562 and Molt-4 respectively.

### Peptides

Clinically tested HLA-A*02:01-restricted LAA peptides (Table 2) had been previously identified from overexpressed genes in leukemia patients compared to health individuals, by reverse immunology using computer based T cell epitope prediction programs such as BIMAS^60^, SYFPEITHI^61^, or artificial neural networks (ANNs) in the immune epitope database (IEDB)^62^. These peptides together with new HLA-A*02:01-restricted LAA peptides identified from MUTZ3 DCs and THP1MФ HLA class I peptidomes (Table 1) together with a HIV polymerase peptide were synthesized with a purity >95% by EMC microcollections GmbH (Tübingen, Germany). DMSO (Pierce, Rockford, Illinois, USA) was used to dissolve the lyophilized peptides before storage at −20 °C.

### T2 cell line HLA-A*02:01 binding assay

To determine the HLA-A*02:01 binding of the peptides in Tables 1 and 2 the TAP-deficient HLA-A*02:01-positive T2 lymphoma cell line was used as previously described^63^ with minimal modifications detailed in Supplementary Methods. The P564-HIVpol peptide LLFGXPVYV and T2 cells without peptide was used as positive and negative control respectively.

### Flow cytometric analysis of CD8+ T cells frequencies

To determine the CD8+ T cells frequencies in the PBMC from a healthy donor and CML patients, pre and post peptide-primed PBMCs were stained with fluorochrome-labeled monoclonal antibodies against CD8 (BD Bioscience, Heidelberg, Germany). The frequencies of cells expressing this marker was determined with a FACS Calibur flow cytometer (Becton Dickinson, Heidelberg, Germany). CellQuest (Becton Dickinson, Heidelberg, Germany), and FlowJow (FlowJo LLC, Oregon, USA) software were used to process and analyze the data, respectively.

### LAAs immunogenicity

To determine the immunogenicity of the LAAs, the NetMHCpan 4.0 algorithm in IEDB^64^ was used. The immunogenicity was determined for HLA-A*01:01, HLA-A*02:01, HLA-A*11:01, HLA-A*24:02, HLA-C*06:02, HLA-C*07:01 and HLA-C*07:02, individually and all combined. These alleles together are expressed in 90% of the population^65^. IC_50_(500) nM binding affinity threshold was used as threshold for immunogenicity, and immunogenicity scores were presented as 1/IC_50_(500) nM.

### LAA gene expression in CML, AML, normal hematopoietic cells and tissues, and protein interaction partners

To determine the gene expression profiles of the LAAs in CML, AML, normal human hematopoietic cells and other major human tissues, as well as protein interaction partners, the bloodspot database^50^ (a database of gene expression profiles for healthy and malignant hematopoietic cells and tissues), BioGPS (http://biogps.org/) and the search tool for the retrieval of interacting proteins (STRING version 11.0) database^66^ (a database of known and predicted protein-protein interactions) were used respectively. The gene expression profiles were determined using bloodspot based on curated microarray data. The data for human CML and AML were from GSE13159.The samples were derived from bone marrow. The AML were with t(15; 17), inv(16)/t(16;16), t(8; 21), t(11q23)/MLL and had complex aberrant karyotypes, and analysed with Affymetrix HG-U133 Plus 2.0 GeneChips^67,68^. The data for human normal hematopoietic cells were from GSE42519, and derived from 34 healthy donors hybridized to Affymetrix HG-U133 Plus 2.0 GeneChips^69^. The gene expression profiles of normal major human tissues was determined using BioGPS (http://biogps.org/). The data are from gene ATLAS U133A, gcrma, derived from 79 human samples hybridized to Affymetrix HG-U133A gene chips^70^. A low gene expression intensity cutoff of 10% was used. STRING version 11.0 was used to determine known eLAAs and pLAAs protein interaction partners, experimentally determined from various biochemical, biophysical and genetic techniques. A medium interaction score of 0.400 was applied with a cutoff of 10 interaction partners at primary level against *Homo sapiens*.

### Statistical analysis

Paired student’s t-test was used to compare LAA gene expression for human CML and AML cells and the highest expression in normal human hematopoietic cells. The differences were indicated as significant when *p < 0.05.

## Supplementary information

Supplementary Materials

## Data Availability

For original data and detailed protocols, please contact peter.walden@charite.de. The peptidome data are deposited in the Immune Epitope Database (IEDB) at https://www.iedb.org/, Dataset identifier 1030722.
