# Supplementary material for: HLA class I-restricted T cell epitopes isolated and identified from myeloid leukemia cells
Source: Sci Rep. 2019 Oct 1;9:14029. doi: 10.1038/s41598-019-50341-7 (PMC6773711; doi:10.1038/s41598-019-50341-7)

HLA class I-restricted T cell epitopes isolated and identified from myeloid leukemia cells

Lydon Wainaina Nyambura[1], Alejandro Azorin Muñoz[1], Philipp le Coutre[2] and Peter Walden[1*]

Supplementary Methods

MUTZ3 and THP1 cells, and differentiation to DCs and macrophages

The acute myeloid leukemia cell line MUTZ3 (DMSZ GmbH, Braunschweig, Germany) was cultured in α-MEM medium (Life Technologies, Grand Island, NY), supplemented with 20% heat-inactivated fetal calf serum (Biochrom, Berlin, Germany), 10% conditioned medium from the bladder carcinoma cell line 5637, 1% penicillin/streptomycin (Invitrogen, Karlsruhe, Germany), and 50 μM β-mercaptoethanol at 37°C in 8% CO_2. The acute monocytic cell line THP1 (ATCC TIB-202) were cultured in RPMI 1640 medium (Invitrogen, Karlsruhe, Germany) supplemented with 10% heat-inactivated fetal calf serum and 1% penicillin/streptomycin at 37°C under 8% CO_2. Differentiation of MUTZ3 to MUTZ3-derived immature dendritic cells (MUTZ3 iDC) was achieved by culturing for 7 days in the presence 20 ng/ml recombinant human IL-4 (Peprotech, Rocky Hill, NJ), 100 ng/ml recombinant human GM-CSF (Genzyme, Cambridge, MA), and 2.5 ng/ml recombinant human TNF-α (Strathmann Biotech, Hamburg, Germany) without the conditioned medium from the 5637 cell line, and refreshing the cytokines at day 3. Further differentiation to MUTZ3-derived mDC (MUTZ3 mDC) was achieved by addition of 10 ng/ml LPS (Sigma-Aldrich, Munich, Germany) at day 7 and culturing for additional 48–72h. THP1 macrophage (THP1MΦ) was generated by differentiating THP1 cells in the presence of 50 ng/ml phorbol 12-myristate 13-acetate (PMA) (Sigma, Steinheim, Germany) for 48h.

*T2 cell line HLA-A*02:01 binding assay*

2 x 10^5/ml T2 cells were seeded in DMEM (Gibco-BRL, Karlsruhe, Germany) with 2 μg/ml β_2-microglobulin (Sigma-Aldrich, Steinheim, Germany) and incubated with 100 μM peptides for 18

h at 37°C and 8% CO_2. After incubation, cells were harvested by centrifugation at 400 x g for 7 min, washed with PBS (Gibco, Grand Island, NY,USA) re-suspended in 200 µl PBS (Gibco, Grand Island, NY,USA) and incubated with the anti-human HLA-A2 FITC-labelled BB7.2 mAb (BioLegend, Eching, Germany) for 45 min at 4°C. Cells were then washed with 500 µl PBS and re-suspended in 400 µl PBS. Fluorescence intensity was measured using FACS Calibur flow cytometer (Becton Dickinson, Heidelberg, Germany), and the data were processed and analyzed with CellQuest (Becton Dickinson, Heidelberg, Germany) and WinMDi 2.9 (Purdue University, USA) softwares.

IFNγ ELISpot Assay

2.5 x 10^5 PBMCs from healthy donors and a CML patient were pulsed individually with 10µg/ml of each peptide in ExVivo 15 serum-free medium (Biowhitaker, Belgium) in 96 well multiscreen plates (Milipore, Darmstadt, Germany) coated overnight at 4°C with 100 µl (1:1,000) of anti-human INFγ capture mAb (Endogene, Pierce Biotechnology, Inc). PBMCs with culture medium only were used as negative control. The ELISpot plates were incubated for 18h hours at 37°C and 8% CO_2, then washed twice with PBS, and incubated with 50 µl (1:500) biotinylated anti-human IFNγ antibody (Endogene, Pierce Biotechnology) for 2h at room temperature (RT). Following washing twice with PBS, the plates were incubated with 50 µl streptavidin-conjugated alkaline phosphatase (1:2000) (Roche, Mannheim, Germany) for 1h at RT. Thereafter, the plates were washed 3 times with 100 µl PBS followed by 50 µl BCIP/NBT (5-Bromo-4-chloro-3-indolyl phosphate/Nitro blue tetrazolium) substrate for 30 min as per manufacturer's instructions (Moss Inc., Pasadena, CA, USA). ELISpot plates were dried overnight at 4°C and thereafter scanned and counted using Bioreader 3000 (BioSys, Karben, Germany).

SUPPLEMENTARY FIGURE 1

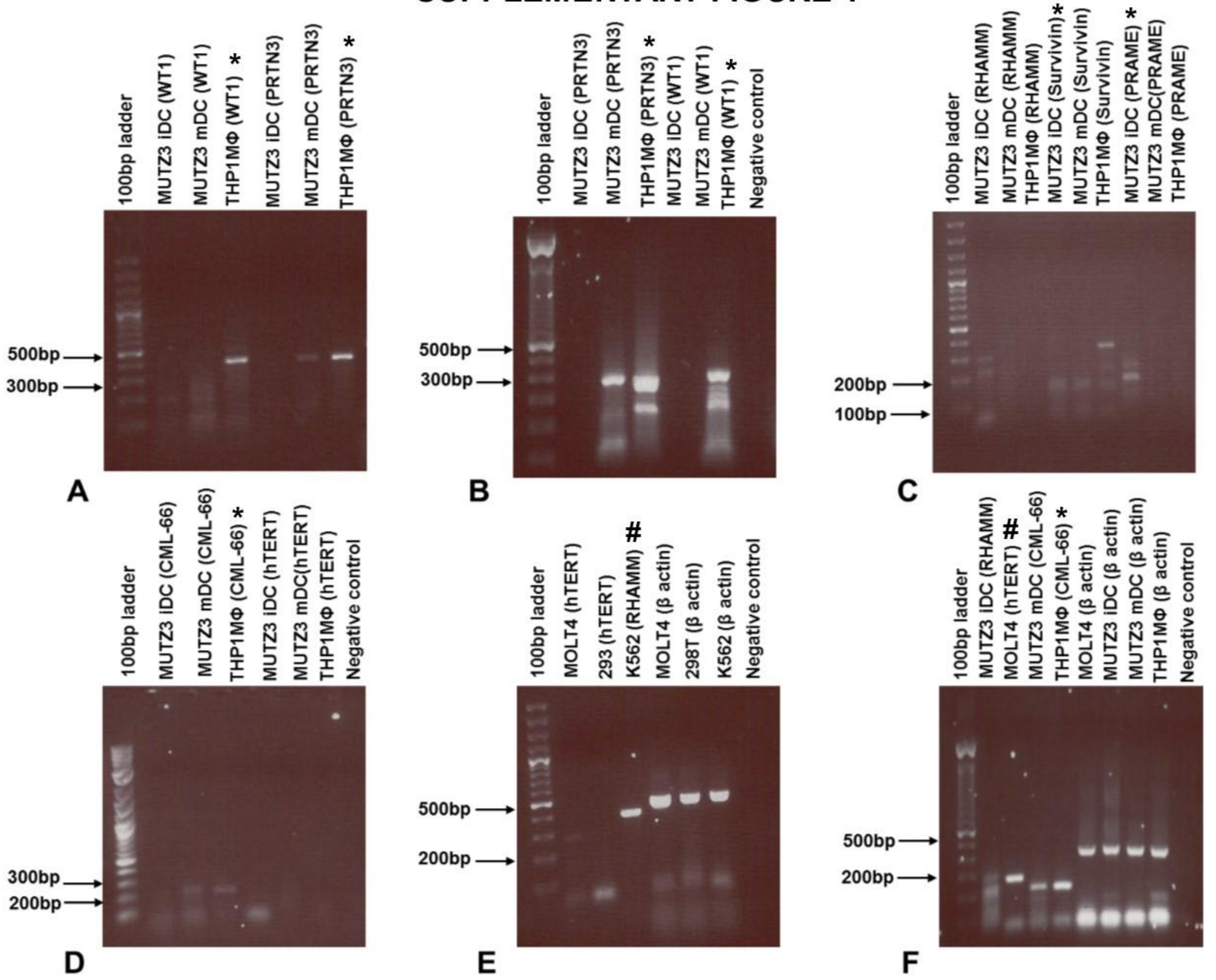

* also as a positive control; # positive control

Supplementary Figure 2

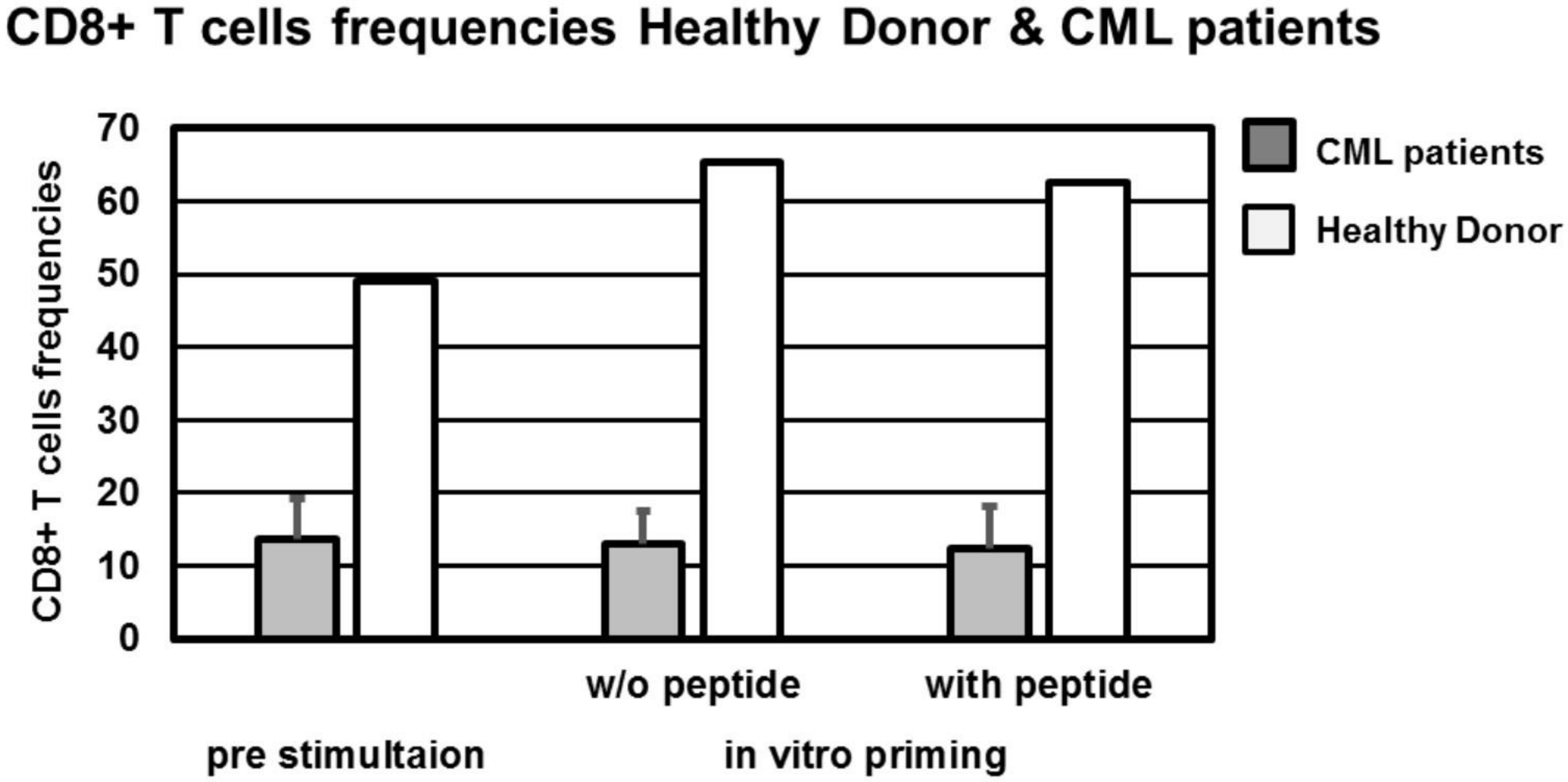

Supplement: Supplementary file 1 — Supplementary Materials [file 41598_2019_50341_MOESM1_ESM.pdf]
